# X-ray Photon Counting and Two-Color X-ray Imaging Using Indirect Detection

**DOI:** 10.3390/s16060764

**Published:** 2016-05-26

**Authors:** Bart Dierickx, Qiang Yao, Nick Witvrouwen, Dirk Uwaerts, Stijn Vandewiele, Peng Gao

**Affiliations:** Caeleste CVBA, Hendrik Consciencestraat 1 b, 2800 Mechelen, Belgium; Qiang.yao@caeleste.be (Q.Y.); Nick.witvrouwen@caeleste.be (N.W.); Dirk.uwaerts@caeleste.be (D.U.); Stijn.vandewiele@caeleste.be (S.V.); Peng.gao@caeleste.be (P.G.)

**Keywords:** X-ray photon counting, scintillator, color X-ray, spectral X-ray

## Abstract

In this paper, we report on the design and performance of a 1 cm^2^, 90 × 92-pixel image sensor. It is made X-ray sensitive by the use of a scintillator. Its pixels have a charge packet counting circuit topology with two channels, each realizing a different charge packet size threshold and analog domain event counting. Here, the sensor’s performance was measured in setups representative of a medical X-ray environment. Further, two-energy-level photon counting performance is demonstrated, and its capabilities and limitations are documented. We then provide an outlook on future improvements.

## 1. Introduction

Photon-counting-based X-ray imaging is assumed to be superior in performance as compared to the more state-of-the-art charge integration X-ray imaging [1]. This is obvious at very low fluxes where photon counting yields quantum limited noise, but also, at high fluxes, photon counting has a DQE (detective quantum efficiency) advantage over integration. A second advantage of photon counting, especially for medical imaging, is that it offers the possibility of extracting spectral information from each photon separately, thus without multiple exposures or an increased X-ray dose. This spectral information reflects the chemical composition of the tissue examined—in this case, the ratio of carbon to oxygen [2,3].

Most, if not all of today’s successful photon counting X-ray imagers are based on “direct detection” [4,5,6,7,8,9,10,11,12,13], *i.e.*, the X-ray detection happens by absorption of the photon in a high-Z semiconductor photo diode or photo resistor. From a pure detection performance standpoint, this approach is ideal: the photo-electric conversion happens in a very limited volume, and the energy quantum is deposited in a narrow trace or cloud of secondary electron-hole pairs, which are quickly and with little sideward dispersion collected by the electric drift field. As the collection time is in the order of nanoseconds, the direct detector can be operated at very high count rates. As the secondary charges remain confined, the modulation transfer function (MTF), and hence the DQE, is excellent, and the reproducibility of the collected charge packet size is nearly perfect, allowing, if required, an accurate photon energy measurement.

The limiting factor for the widespread use of direct detection in photon counting imaging is the cost and manipulation of the material.

The alternative route, indirect detection, *i.e.*, detection of the X-ray photon indirectly by first absorbing it in a high-Z scintillator and then detecting the secondary, visible light radiation by a visible light image sensor, is economically viable [14]. Many scintillators are inexpensive and easy to co-integrate, and CMOS visible-light event counting is an easily scalable and mature technology. However, indirect detection has disadvantages as compared to detect detection: the overall indirect process has a significantly lower photon to electron conversion, suffers from slow decay times, may suffer light dispersion and poor MTF, and has poor reproducibility of charge packet sizes, making photon energy measurements unreliable.

In this paper, we report on our experiments to design, manufacture, and evaluate a two-energy-channel indirect detection X-ray image sensor [15,16,17,18]. We prove its feasibility, demonstrate that decent performance can be reached in pure counting and in energy resolution, and also show which limitations are present. At the end of the paper, we provide an outlook on a route for future improvements and solutions for the shortcomings.

## 2. Design of a Photon Counting Image Sensor

**Figure 1:**
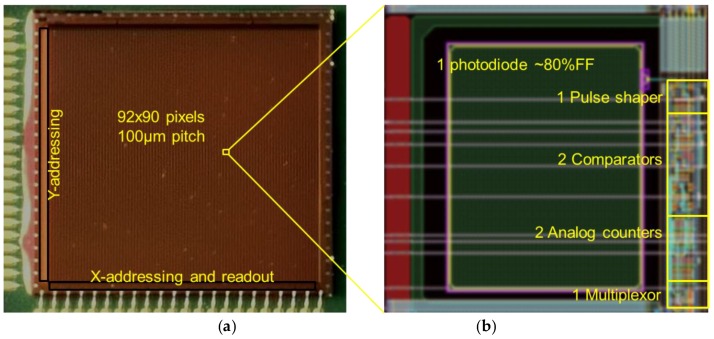
(**a**): microphotograph of the QX2010 device wire bonded on the CoB (chip on board); (**b**): Layout detail of one pixel, indicating the relative sizes and positions of the main pixels parts.

Our experimental device “QX2010” (Figure 1 and Table 1) is an image sensor with an array of X-ray photon counting pixels based on indirect (*i.e.*, using a scintillator) X-ray detection. Pixels are capable of detecting charge packets of at least 100 electrons per X-photon. This requirement stems from practice: this is the amount of charge carriers collected by the visible light photodiode for a single event, for photon energies in the range of medical X-ray, *i.e.*, 20 to 100 keV, using typical scintillator materials and scintillator thicknesses for medical X-ray imaging.

### 2.1. Pixel Topology

**Figure 2:**
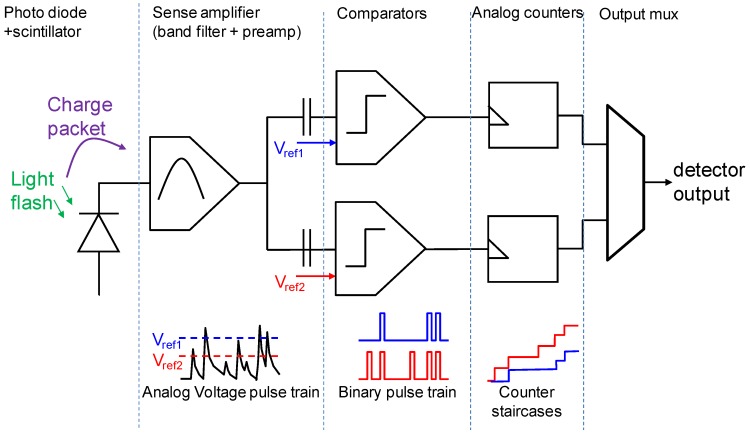
Topology of the QX2010 pixel.

**Figure 3:**
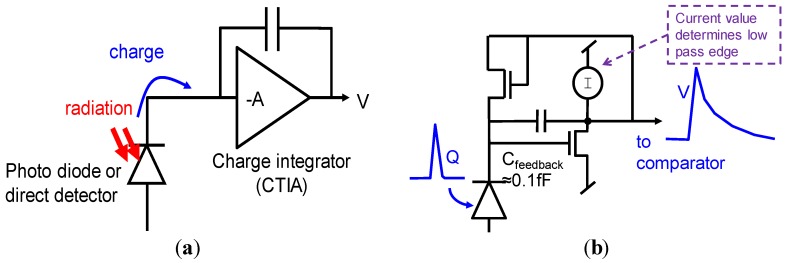
(**a**): General concept of a charge sensitive “charge trans-impedance amplifier” (CTIA); (**b**): CTIA with resistive feedback implemented as a MOSFET, becoming a “pulse shaper.”

The pixel topology is shown in Figure 2. It is essentially a two-channel counting pixel. As transistor number and area is expensive, we had to reduce the complexity of each component to the minimum. The concept of the charge packet sense amplifier or “pulse shaper” is shown in Figure 3.

**Figure 4:**
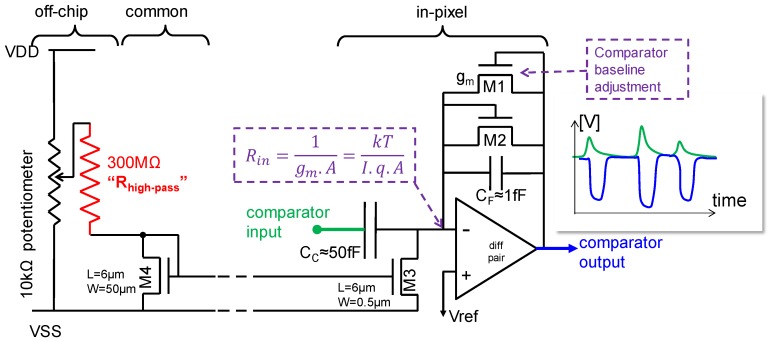
Comparator concept.

**Figure 5:**
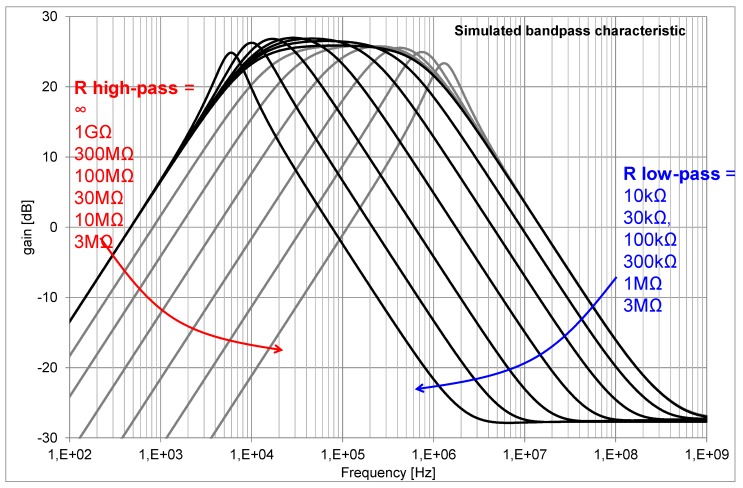
Simulated bandpass characteristics of the combination of pulse-shaper and comparator.

The comparator concept of this pixel is shown in Figure 4. The single pulse shaper feeds two comparators, each having different threshold “Vref.” Both the bias currents in the pulse shaper and comparators are set through current mirrors. By choosing a proper value for the off-chip bias resistors of these current mirrors, one can independently set the overall high pass and low pass edge of the band-pass characteristic of the combination pulse-shaper + comparator, as exemplified in Figure 5.

**Figure 6:**
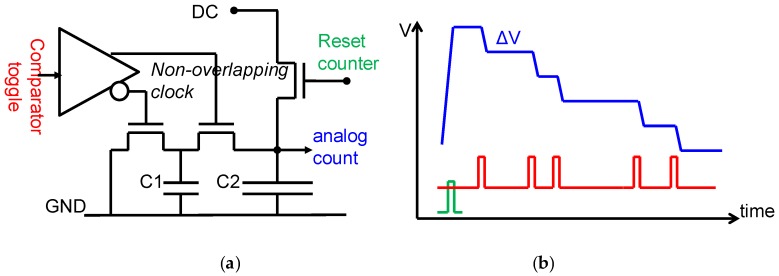
Analog domain counter. (**a**): circuit concept; (**b**): signals as expected on key nodes of the circuit concept.

**Figure 7:**
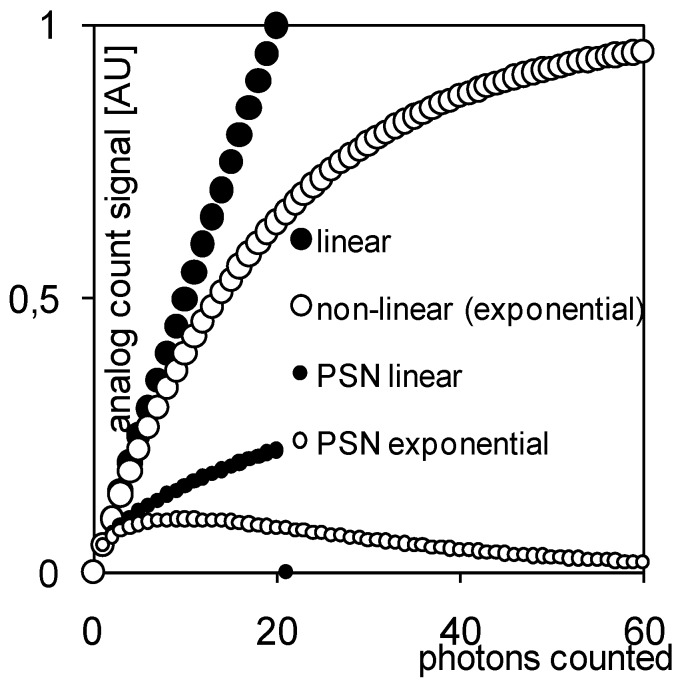
“Non-linear analog counting” is valid as long as the voltage step can be discriminated by the external ADC; yet this may be relaxed as long as the PSN (photon shot noise) expressed in voltage steps can be discriminated by the ADC. PSN is calculated as the square root of the number of photons counted.

An important feature of the pixel is the introduction of what we call “non-linear analog domain counting.” A classic, digital domain counter consisting of logic gates such as DFFs and NANDs would result in a sub circuit containing hundreds of transistors. This would not only eat up the available area in the pixel, it would also result in poor yield if the pixel array size would be scaled to the size desired in X-ray imaging, being several cm^2^ up to wafer-scale. The solution that we proposed in [15,16] is to replace the digital counter by a circuit that realizes an analog signal staircase, which one may interpret as an extreme example of multi-level logic. Such a circuit can be realized in a very compact fashion, as in Figure 6. Here, an initial DC voltage on the large capacitor C2 is gradually and step-wise decreased by the switched capacitor network around the capacitor C1. Such an approach is acceptable as long as one can unambiguously retrieve the digital count by converting the staircase signal with an analog to digital convertor (ADC), *i.e.*, as long as the steps of the staircase are sufficiently large. As the step height is proportional to the “analog count” value itself, the step height decreases as the number of events increases, in a decaying exponential fashion. The non-linear transfer function of the analog counter helps to extend the range of the counter (Figure 7).

Using such a counter and other, equally compact sub-circuits, we managed to keep the MOSFET circuit area below 20% of the area of the 100 × 100 µm^2^ pixel, leaving almost 75% fill factor for the optical photodiode.

### 2.2. Pixel Measurements with Visible Light

**Figure 8:**
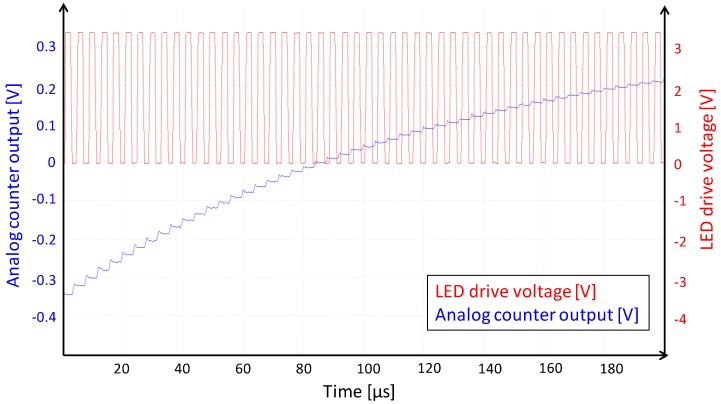
Signal of an analog counter during illumination of the QX2010 by a pulse train of LED pulses. X-axis: Time 0–1 ms; Y-axis: *Red trace* LED forward bias voltage; *Blue trace* analog counter voltage, measured real-time inside the circuit.

The X/Y addressing structure of the QX2010 imager allows us to observe each analog counter in real-time. Figure 8 shows the analog counter response for a periodic visible light LED pulse train.

**Figure 9:**
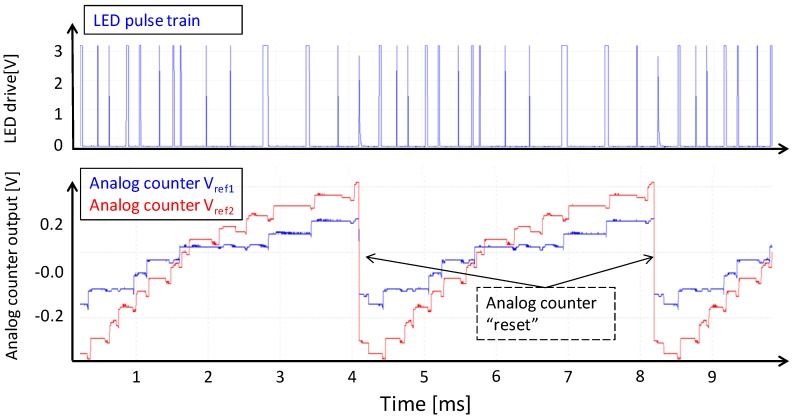
Demonstration of analog counting, emulating the variation of charge packet size by means of LED pulse width modulation. For the evaluation of noise and reproducibility, we defined a repeated random pattern of long and short LED pulses (blue top trace). The bottom traces are the two real-time observed analog counter outputs (with different Vref thresholds) of one pixel.

In Figure 9, we show the response of the analog counter on a pseudo-random LED pulse train. Short pulses create small charge packets, and wide LED pulses create large charge packets, thus mimicking the situation of an X-ray illuminated scintillator that would output a random population of stronger and weaker secondary light flashes. Of interest here is that we clearly see the effect of a different reference threshold voltage of each comparator; we also see a pretty good reproducibility of the counter pattern over time.

## 3. Experimental Results under X-ray Illumination and Discussion

**Figure 10:**
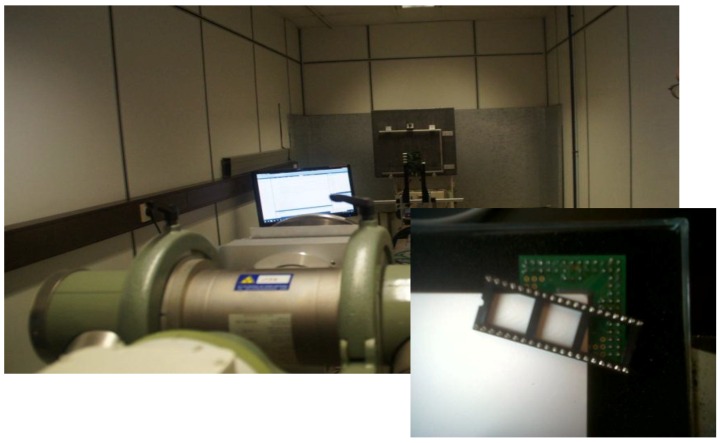
Photograph of an in-beam measurement setup.

In this paragraph we report on the experimental results of the QX2010 device with a CsI scintillator in an X-ray beam. A picture of one of the setups and a detail of the QX2010 device is shown in Figure 10.

### 3.1. Beam Experiments with the QX2010

**Figure 11:**
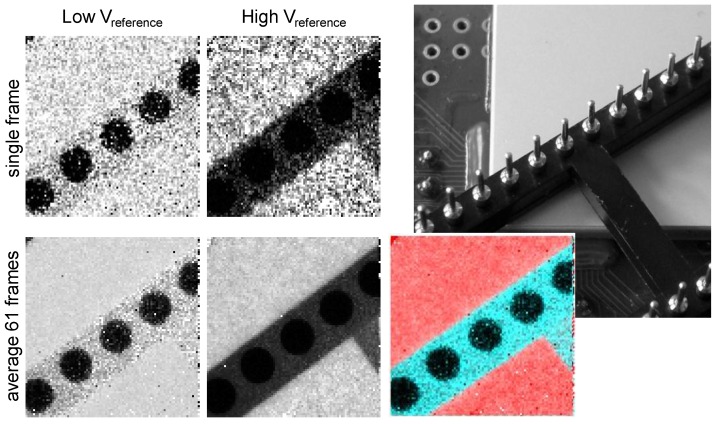
Recorded 92 × 90 pixel images of the object shown at the top right (a DIL socket on top of the scintillator glued in the QX2010). Top left/middle: simultaneous low reference and high reference threshold images from the QX2010. Bottom left/middle: the same, averaged 61 frames to smooth the photon shot noise. Color image: A “color-matrixed” combination of the two bottom left/middle images.

In such experiments, we recorded images under widely different conditions of beam voltages and current—various objects, various bias conditions of the QX2010, and various reference threshold voltages. It is beyond the scope of this paper to report on these. As an example of what such a photon counting imager can do, we show Figure 11. These are very low flux images with on average 10 and 30 counts (high/low threshold) per frame in the brightest parts of the image. In both images, metal parts have very low transmission, but the plastic part shows a distinct difference in transmission. These two “black and white” images can be combined in a color image for rendering.

**Figure 12:**
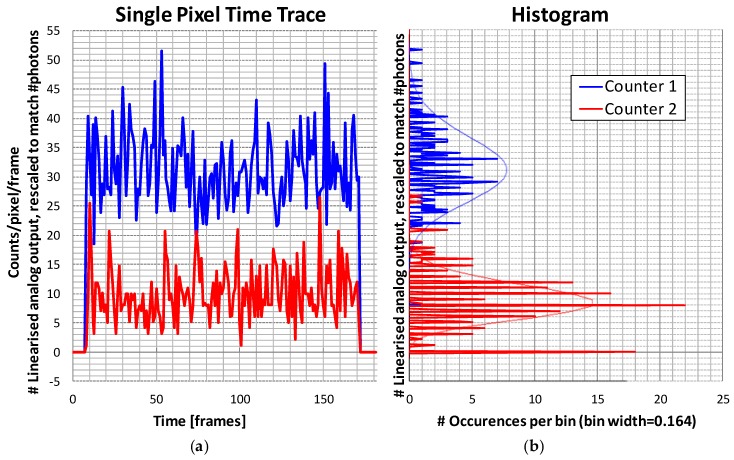
One pixel, two thresholds, observed over a time comprising 180 frames. Beam powers on at frame 9 and powers off after frame 170. (**a**) Counts *versus* frame number; (**b**) Horizontal histogram of the occurrences of analog count values. Thin red and blue lines are theoretical Poisson distributions with the same average counts.

As two count values per pixel per frame are recorded, one can plot these as a series for a certain pixel over time. Such a series is shown in Figure 12. The non-linear analog counter values are properly linearized and scaled on the Y-axis, so that they correspond as closely as possible with the discrete integer counts. One clearly sees the photon shot noise in the X-ray detection. The histogram on the right is compared to a theoretical Poisson distribution with the same average count.

**Figure 13:**
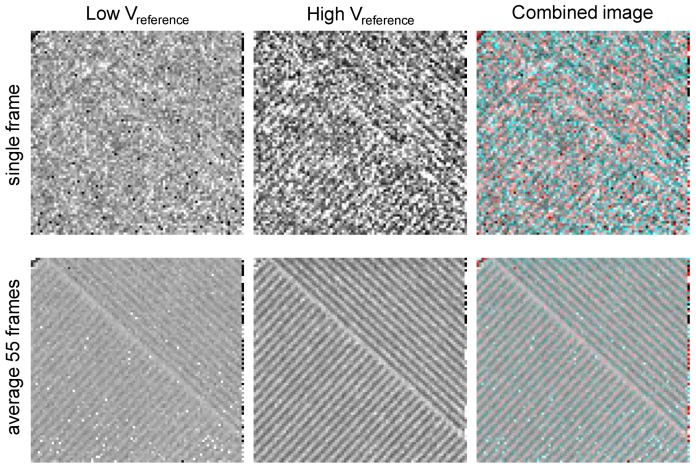
Images of a lead resolution target. Simultaneously recorded frames with two threshold levels (left, middle). Right: “color matrix-like” linear combination of the two frames for color rendering. Upper row: single frames; lower row: average of 55 frames.

Figure 13 displays images taken with a lead resolution target. When recording images of the target with two thresholds, one observes that the image with the higher reference voltage (V_reference_) threshold, *i.e.*, the one recording the largest charge packets only, is sharper and thus has a better MTF. It also has a distinctly lower average count than the lower threshold image. This cannot be explained by any spectral sensitivity effect, as the lead in the target has a practically 100% absorption; thus, both images should be identical. The explanation of this observation is given in the next paragraph.

### 3.2. Discussion on Issues Found

**Figure 14:**
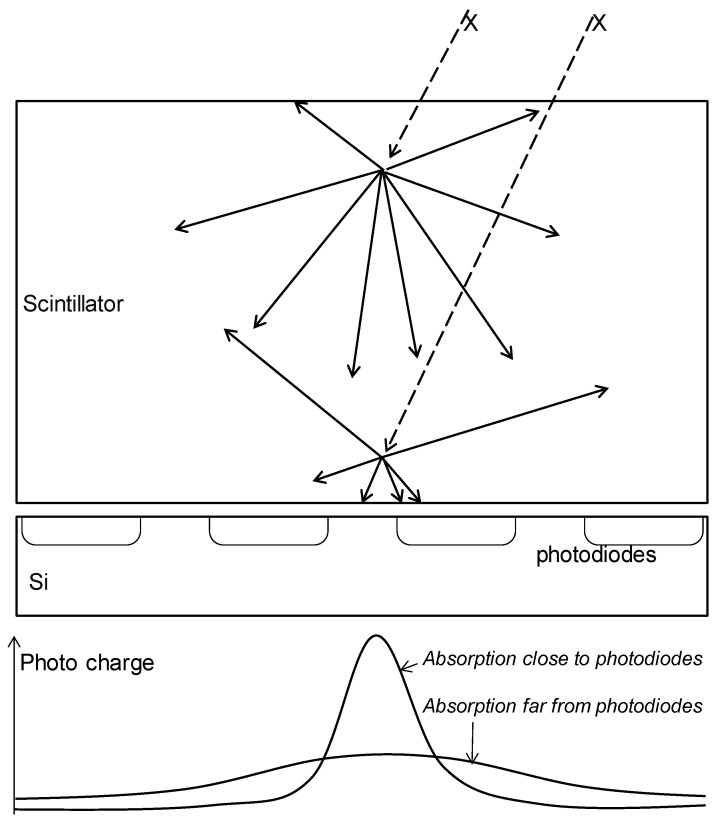
Schematic drawing of Lubbert’s effect in a scintillator-covered Si pixel array.

In these experiments, we demonstrated that scintillator-based photon counting is feasible. At the same time, however, we were able to pinpoint the major shortcomings of scintillator-based photon counting, all related to the well-known *scintillator depth effect* or to *Lubbert’s effect* [19]: the amount of secondary radiation that falls on a single Silicon pixel depends significantly on the depth in the scintillator where the photo-electric conversion from X-ray photo to secondary light flash takes place, as illustrated in Figure 14.

*Shallow* absorption (far from the silicon photodiodes) *versus* *deep* absorption (close to the silicon photodiodes) results in larger or lesser optical diffusion of photo charges to neighboring pixels. As a consequence, one will see

- that the MTF depends on the depth of absorption and hence on the energy of the photon;
- that the number of visible photons per event per pixel depends on depth of absorption, therefore resulting in missed counts if the photo charge is diluted too much;
- that double or multiple (false) counts may occur if the event acts on multiple pixels;
- that these combined effects affect the DQE adversely; and
- that the effect deteriorates the assumed spectral energy sensitivity: the *observed* larger charge packets are not only charge packets originating from higher energy photons, but also X-ray photons absorbed close to the Si. The observed smaller charge packets may as well come from the higher energy photons that are absorbed far from the Si.

## 4. Next-Generation Devices

In this paragraph, we will discuss solutions to the issues encountered, as may be implemented in future devices.

### 4.1. Scintillators with Optical Confinement

**Figure 15:**
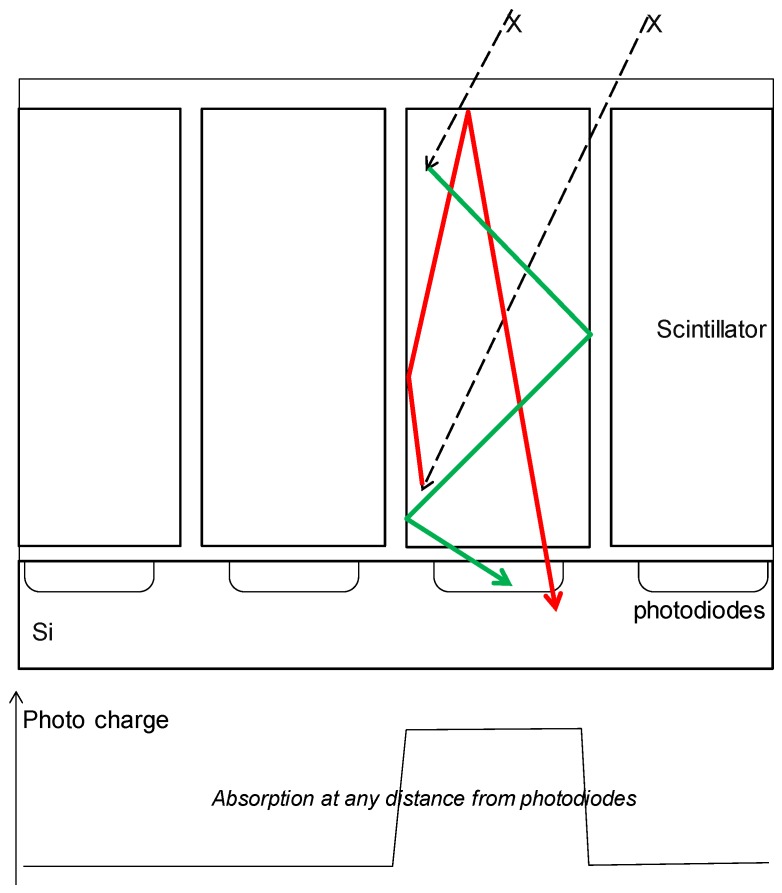
Similar drawing as Figure 14, now with optically confined scintillators on top of the silicon pixels.

Although we see a clear advantage of CsI *versus* most other scintillators, the anisotropic nature of CsI crystals is far from sufficient to cancel Lubbert’s effect. The root cause solution of the light diffusion problem may be in the optical confinement of the light in a volume aligned with the pixel [20,21]. By confining the light in a volume that is equal in size to, or smaller than, the pixel (Figure 15), one will prevent the MFT degradation and thus rival the MTF performance of direct detection.

### 4.2. Combining Photon Counting and Charge Integration in One Pixel

Two other weak points of photon counting in general, and analog domain photon counting specifically, are the limitations of count range and count speed. This also limits the more widespread use of photon counting X-ray imagers.

The count range may be virtually unlimited in digital domain, as long as the number of bits in the counter is high enough. In the analog domain, our concept allows us to reliably count up to about 100, and optimizations might result in counts up to 1000.

The count rates are limited by circuit performance, circuit power, and scintillator decay times. Failure here may lead to missed counts or even to counter paralysis.

Both issues are not present in the classic integrating X-ray imager. It has been proposed before by several groups and users to combine both concepts. In this paragraph, we propose a pixel concept that realizes photon counting and charge integration in the same pixel, on the same photo charge. 

**Figure 16:**
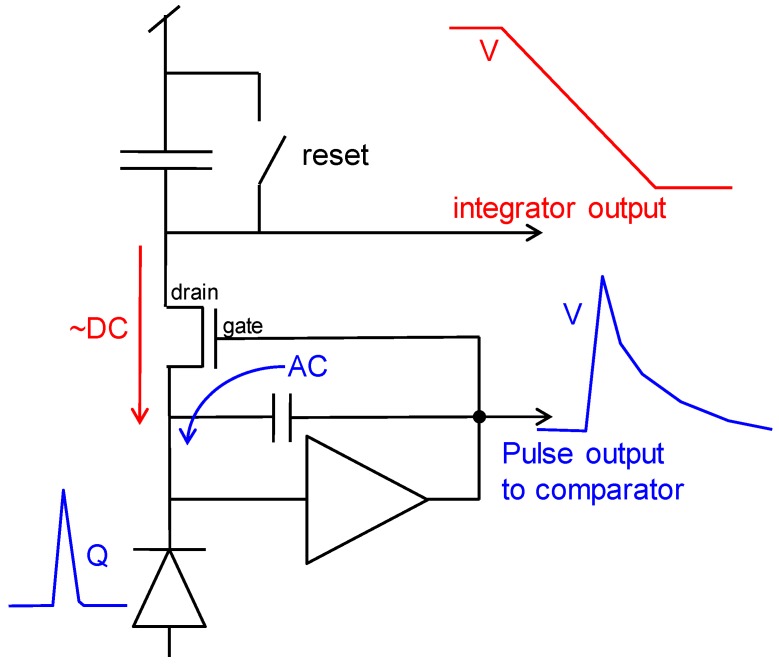
Concept circuit of a pulse shaper that feeds its photocurrent to a charge integrator.

Consider the pulse shaper circuit of Figure 3. In this circuit, both the AC and DC photo currents from the photodiode end up in the output node of the feedback amplifier. By separating the drain and gate of the feedback MOSFET as in Figure 16, one can maintain the pulse shaping properties and add the capability of integrating the DC photo current with a capacitor.

**Figure 17:**
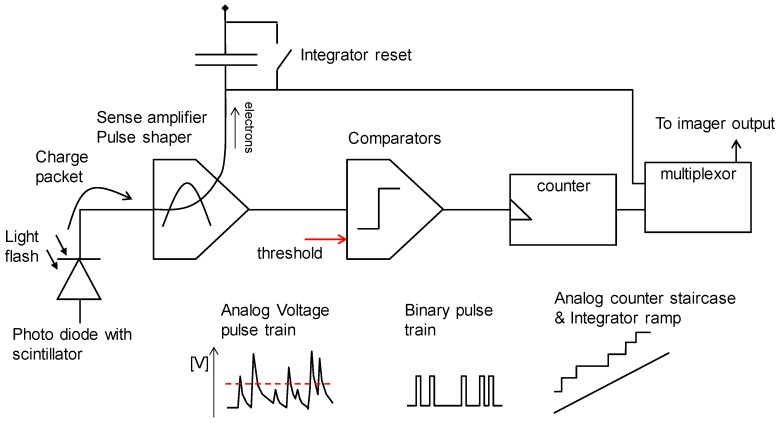
Overall pixel topology allowing simultaneous photon counting and charge integration.

This integrator in itself reminds us of the well-known integrating 3T pixel. The extra circuitry compared to the photon counter is small. The overall pixel may look like the illustration in Figure 17.

This pixel is significantly more compact than the two-channel pixel of Figure 2. Notwithstanding, it has clear advantages:

- It does genuine photon counting. Its low count range and count rate make it suitable for low flux applications such as fluoroscopy. At medium fluxes, this signal may suffer from counter paralysis.
- It does genuine charge integration and can handle any charge quantity as required in medical imaging, limited only by the integration capacitor. These are applications such as mammography and high SNR imaging. In low flux conditions, the charge integration signal will be read noise limited and thus perform worse than the photon counting signal.
- There is a flux range where both signals are available and of good quality. In that range, the ratio of the two signals is a form of spectral information, as treated hereafter.

One can express both pixel signals as voltages in this “pseudo code”:

(1) $$V_{counter}=\#photons∙V_{step}\;\text{and}$$

(2) $$V_{integrator}=\sum_{all\;photons}LO$$

where V_counter_ is the linearized and normalized analog counter signal, V_integrator_ is the offset corrected integrator signal, #photons is the number of photons absorbed during the integration time, V_step_ is the analog counter normalized voltage step, QE is the photodiode’s quantum efficiency at the emission wavelength of the scintillator; the summation is over all primary photons; LO is the light output expressed as secondary photons for each primary photon, which, in good approximation, is proportional to the photon energy hν:

(3) $$V_{counter}\sim \sum_{all\;photons}\left(1\right)\;and$$

(4) $$V_{integrator}\;\sim \sum_{all\;photons}\left(h\nu \right)$$

where α is the proportionality factor between photon energy and the effective number of photoelectrons in the photodiode.

(5) $$\frac{V_{integrator}}{V_{counter}}\sim \frac{\sum_{all\;photons}\left(h\nu \right)}{\sum_{all\;photons}\left(1\right)}\;and$$

(6) $$\frac{V_{integrator}}{V_{counter}}\sim \bar{h\nu }$$

Hence, ratio (Equation (6)) is roughly proportional to the average photon energy during this integration time. The ratio between the counting channel and the integration channel represents the average photo charge per X-photon, which is a direct measure of the average photon energy on that pixel during the integration time and hence contains spectral information relating to the chemical composition of the tissue being imaged.

### 4.3. Photon Shot Noise-Free Spectral Information

A peculiar property of the combination of photon counting and charge integration on the same photo charge is that the above ratio (Equation (6)) is free of photon shot noise. The ratio, even in the hypothetical case of a single (X-ray) photon integrated, yields the energy of that photon.

## 5. Conclusions and Outlook

In this paper, we demonstrated that it is feasible to realize an X-ray photon counting image sensor using indirect detection. The presumed hurdles can all be overcome: the low light output of scintillators is not a showstopper. For the scintillators studied, GOS and CsI, charge packets in the order of 100 to 500 electrons for medical X-ray energies, are perfectly detectable and can be discriminated with low error rates, at least if there is no dilution by optical crosstalk. Although we experienced that in practice, the decay time of the scintillators is not as short as claimed by the suppliers or in textbooks. The actual delay times of CsI are suitable for count rates up to about 100,000 kHz. We managed to design 100-µm pitch pixels with a fill factor close to 75% by using very compact electronics, including “analog domain” counters. 

### Outlook

We expect the concept herein to be scalable to pixel sizes smaller than 50 µm and to array sizes that may go up to the full wafer size. Alternative and variant concepts may reach even smaller pixels pitches by using smaller linewidth CMOS technologies, or by using backside illumination, so that the fill factor remains high notwithstanding a small photodiode junction surface, or hybrid configurations of the indirect and even direct detection type.

We pointed out that a combination of photon counting and charge integration on the same photo charge expands the usability of the concept; an interesting side effect is the capability of obtaining photon shot noise—free spectral information.

Concerning the basic performance limitations due to Lubbert’s effect or the scintillator absorption depth effect, we expect that the root cause solution must come from CMOS-process compatible optical confinement of the scintillator’s emitted light inside the boundaries of the pixel.

## Figures and Tables

**Table 1:** QX2010 image sensor specifications.

Item	Specification	Item	Specification
Technology	TowerJazz TSL018IS	Detection concept	Photon counting with a scintillator
Die size	1 × 1 cm	Sense node capacitance	2…3 fF
Array size	X: 91 + 1 test column	Wavelength spectrum	400…900 nm (typical Si)
	Y:90		
Pixel pitch	100 µm	QE × FF (quantum efficiency × fill factor)	~50% assuming optical glue between scintillator and image sensor
Analog counter step height	20 mV, exponentially decaying (see further)	FF (Fill factor)	75%, metal limited
Number of energy channels	2	Smallest charge packet that can be counted	~50 electrons estimated
Test pixels	In column 92	Q_N_ noise on threshold	~15 e-_RMS_ estimated
Acquisition scheme	Global shutter (*i.e.*, global reset of counters)	Q_N_ variability	Not measured
Array readout scheme	X/Y addressing	Dark current and dark current variability	Not considered (DC current does not affect a pulse shaper)
#transistors per pixel	45 (53 in some test pixels)	MTF	Not measured or not relevant on imager part only.
Full frame readout time	8 ms Most measurements done with frame time, including count time 80 ms.	FPN (threshold voltage accuracy & reproducibility)	15 e-_RMS_ estimated
Maximum count rate (separating two events)	~1000 kHz max. Most measurements with setting allowing up to 100 kHz.	PRNU (photo response non-uniformity)	No data
Pulse shaper band	Adjustable by current mirrors	Threshold of comparators	Adjusted by voltage
Number of IO pins	40 at two edges	Power consumption at 5 fps	30 mW
